# The Therapeutic Relationship and Adherence to Antipsychotic Medication in Schizophrenia

**DOI:** 10.1371/journal.pone.0036080

**Published:** 2012-04-27

**Authors:** Rosemarie McCabe, Jens Bullenkamp, Lars Hansson, Christoph Lauber, Rafael Martinez-Leal, Wulf Rössler, Hans Joachim Salize, Bengt Svensson, Francisco Torres-Gonzalez, Rob van den Brink, Durk Wiersma, Stefan Priebe

**Affiliations:** 1 Unit of Social and Community Psychiatry, Queen Mary University of London, London, United Kingdom; 2 Central Institute for Mental Health, Mannheim, Germany; 3 Department of Health Sciences, University of Lund, Lund, Sweden; 4 Department of Psychiatry, University of Liverpool, Liverpool, United Kingdom; 5 Intellectual Disability-Dual Diagnosis Research Unit, Fundación Villablanca, Reus, Spain; 6 Department of General and Social Psychiatry, Psychiatric University Hospital, Zurich, Switzerland; 7 Department of Psychiatry, University of Granada, Granada, Spain; 8 Department of Psychiatry, University of Groningen, Groningen, The Netherlands; University of Manitoba, Canada

## Abstract

**Objective:**

Previous research has shown that a better therapeutic relationship (TR) predicts more positive attitudes towards antipsychotic medication, but did not address whether it is also linked with actual adherence. This study investigated whether the TR is associated with adherence to antipsychotics in patients with schizophrenia.

**Methods:**

134 clinicians and 507 of their patients with schizophrenia or a related psychotic disorder participated in a European multi-centre study. A logistic regression model examined how the TR as rated by patients and by clinicians is associated with medication adherence, adjusting for clinician clustering and symptom severity.

**Results:**

Patient and clinician ratings of the TR were weakly inter-correlated (r_s_ = 0.13, p = 0.004), but each was independently linked with better adherence. After adjusting for patient rated TR and symptom severity, each unit increase in clinician rated TR was associated with an increase of the odds ratio of good compliance by 65.9% (95% CI: 34.6% to 104.5%). After adjusting for clinician rated TR and symptom severity, for each unit increase in patient rated TR the odds ratio of good compliance was increased by 20.8% (95% CI: 4.4% to 39.8%).

**Conclusions:**

A better TR is associated with better adherence to medication among patients with schizophrenia. Patients' and clinicians' perspectives of the TR are both important, but may reflect distinct aspects.

## Introduction

Adherence to treatment in schizophrenia is generally regarded as central for optimizing recovery [1]. However, non-adherence remains a significant clinical problem, with rates of non-adherence approximately 50% [2]. Non-adherence is linked to relapse, rehospitalisation and poor quality of life. Meanwhile, the therapeutic relationship (TR) between patient and clinician has been found to be important for treatment adherence in other psychiatric conditions. The TR has been extensively studied since it was highlighted by Freud [3]. He wrote that “The first aim of the treatment consists in attaching [the patient] to the treatment and the person of the physician”. Since then, the TR has been described as the “quintessential integrative variable” across different forms of psychotherapy [4] and has been consistently found to predict the outcome of therapy [5]. In psychiatric treatment outside formal psychotherapy, the TR is a more global concept [6]. It tends to be used to denote the quality of the whole relationship rather than specific aspects such as the collaborative bond or the transference relationship [6]. Nonetheless, there is increasing evidence that the TR also predicts outcome of complex psychiatric treatment across diagnoses and treatment settings [7]–[14].

Previous research in psychiatry has investigated the predictive role of the TR with respect to so-called distal outcomes of treatment such as symptom change and social functioning. Few studies have investigated whether the TR is associated with proximal treatment outcomes, e.g., engagement and adherence. This is of interest given the suggestion that a causal chain links each outcome measure in a continuum to the next more distal outcome measure [15], e.g. that adherence to medication leads to better symptom levels, and is important for designing interventions to influence specific outcomes in the treatment chain.

In a recent study by Day et al. [16], the TR was found to predict attitudes towards antipsychotic medication. Various studies have found that a more favourable TR is linked with better adherence to medication in other psychiatric disorders, namely depression [9], [17] and bipolar disorder [18]. Moreover, Holzinger et al. [19] found that patient's assessment of the TR was associated with adherence as reported by patients themselves. It remains unclear whether the TR also predicts adherence to medication when adherence is not based on self-report. Since previous research has shown that the clinician and patient perspective on the quality of the TR are not the same in psychiatric treatments [20], both perspectives need to be considered.

### Aim of the study

The aim of this study was to investigate the association between both clinician and patient ratings of the TR and adherence to antipsychotic medication.

## Methods

### Data Collection

Data was collected in the baseline assessment of a randomized controlled trial to evaluate a new intervention to structure patient-clinician communication, DIALOG, described in detail elsewhere [21]. Data were collected before patients began participating in the trial so they were not influenced by the trial protocol. Data were collected between December 2002 and May 2004. Researchers not involved in the patients' care conducted the interviews. Patients were interviewed in the clinical setting or at home according to their preference.

### Setting

The setting was community mental health services in Granada (Spain), Groningen (The Netherlands), London (United Kingdom), Lund (Sweden), Mannheim (Germany), and Zurich (Switzerland) covering urban and mixed urban-rural areas. The number of community mental health teams included per country varied between 2 (Lund) and 6 (London). All teams were multidisciplinary and provided comprehensive care programmes for people with severe and enduring mental illness. They operated a key worker system in which every patient has a designated clinician, i.e. the keyworker, working within a team with lead responsibility for care co-ordination and delivery [21].

### Participants

Clinicians had a professional qualification in mental health or a minimum of one-year professional experience in an outpatient setting, and an active caseload as a key worker. The caseloads of participating clinicians were screened to identify suitable patients meeting the following inclusion criteria: living in the community and treated as outpatients by community psychiatric teams; at least 3 months of continuous care in the current service; capable of giving informed consent; having sufficient knowledge of the language of the host country; a primary diagnosis of schizophrenia or related psychotic disorder (ICD-10 = F20-F29); aged between 18 and 65 years of age; having routinely at least one meeting with their clinician every two months; and having no severe organic psychiatric illness or primary substance abuse. Patients were first informed about the study by clinicians and, if they agreed, approached by a researcher for consent.

### Ethics statement

The study was approved by the following ethics committees: Hospital Universitario San Cecilio Ethics Committee (Granada, Spain), Certified Medical Ethical Committee of the University Medical Centre (Groningen, The Netherlands), East London and the City Health Authority Research Ethics Committee (London, UK), The Research Ethics Committee, Medical Faculty, Lund university (Lund, Sweden), Medizinische Ethik-Kommission II der Ruprecht-Karls-Universität Heidelberg (Mannheim, Germany) and Kantonale Ethikkommission (Zurich, Switzerland).

### Measures

#### Diagnosis

Psychiatric diagnosis was obtained through a standardized, computer based method using operationalised criteria (OPCRIT) [22].

#### Therapeutic Relationship

The TR was assessed with the Helping Alliance Scale, which has a patient (HAS-P) and a clinician (HAS-C) version. Both scales have established reliability and validity [23], [24]. The patient version has 6 questions. Five questions are self-rated on a scale from 0 (not at all) to 10 (entirely): receiving the right treatment, feeling understood, feeling criticized, keyworker committed to and actively involved in treatment, trust in keyworker and his/her professional competence. The sixth question “How do you feel immediately after a session with your keyworker?” has three possible responses: worse, unchanged or better, scored 0, 5 and 10 respectively. The six questions are summed and divided by 6 to yield a mean score (a higher score indicates a better relationship). The clinician version has five questions, self-rated on a scale from 0 (not at all) to 10 (entirely): get along with patient, understand the patient and his/her views, look forward to meeting patient, actively involved in patient's treatment, can help the patient and treat him/her effectively. The scores are summed and divided by 5 to yield a mean score (a higher score indicates a better relationship).

#### Medication Adherence

Adherence with antipsychotic medication over the previous three months was rated using the Buchanan criteria [25] by the clinician in closest contact with the patient. There were 3 possible ratings: 1 = >75%; 2 = 25–75%; and 3 = <25% (a higher score indicates poorer adherence). The rating was based on knowledge of the patient from routine clinical contact. In 78% of cases, collateral information was also used to make the rating: in 49% of cases, this was information obtained from depot, supervised drug intake or drug testing. In a further 29%, this was information obtained from others involved in the patient's care (e.g. pharmacist, general practitioner, family member).

#### Symptoms

Interviewers assessed patients' symptoms on the 30-item Positive and Negative Syndrome Scale (PANSS) [26]. Inter-rater-reliability using videotaped interviews for PANSS was good (Cohen's kappa = 0.71). The scale assesses positive, negative and general symptoms and is rated on a scale of 1–7 (with higher scores indicating more severe symptoms).

Socio-demographic characteristics of patients and clinicians and the time patients had spent in psychiatric treatment were also obtained.

### Statistical Analysis

The association between length of time in treatment and the TR was explored with bivariate correlations (Spearman rho). A one-way ANOVA was used to compare professional background of keyworkers on both keyworker and patient ratings of the TR. The odds of good adherence to medication were compared to the odds of poorer adherence using a logistic regression with standard errors robust to clustering of patients within clinicians. The independent predictors were patient and clinician ratings of the TR and symptoms, as symptoms are known to influence adherence in schizophrenia [27]. Country was also entered into the model. The dependent variable was adherence to antipsychotic medication. The number of patients for whom there was complete data for this analysis was 466. An additional analysis was conducted on the subgroup of patients receiving depot medication as the reliability of assessing adherence in this subgroup is high, i.e., the depot injection either happened or not. The same logistic regression model was applied. This analysis was conducted on 90 patients. Statistical analyses were conducted using SPSS 18.0 for Mac (2010) and Stata 9.2 (2007).

## Results

### Participants

134 clinicians consented to participate, a 74% consent rate. From their caseloads, 507 patients agreed to participate, a 67% consent rate. The number of patients per clinician ranged from 1 to 12, with a mean of 3.73 patients each. 88 patients were recruited in Granada, 99 in Groningen, 99 in London, 61 in Lund, 83 in Mannheim and 77 in Zurich. Sociodemographic and clinical characteristics of participants are presented in Table 1. The mean number of years since patients' first contact with mental health services was 15.6 (SD 10.3).

**Table 1:** Sociodemographic & Clinical Characteristics of Clinicians and Patients.

Clinician Characteristics N = 134	
Age: mean (SD)	43.81 (8.73)
Gender (%) Female	62.6
Male	37.4
Profession (%)	
Psychiatric nurse	48.1
Social worker	22.2
Psychiatrist	10.4
Psychologist	5.2
Other	14.1
Length of service in years: mean (SD)	15.4 (22.0)
Total caseload: mean (SD)	30.7 (59.7)
Therapeutic relationship HAS-Clinician: mean (SD) [range]	7.5 (1.3) [0–10]

Patient Characteristics N = 507	
Age: mean (SD)	42.2 (11.4)
Gender (%) Female	34
Male	66
Time in treatment in years: mean (SD)	15.6 (10.3)
Previous hospital admissions: mean (SD)	5.2 (7.3)
Symptoms: PANSS Positive	14.9 (5.8)
Symptoms: PANSS Negative	16.5 (6.6)
Symptoms: PANSS General	32.3 (9.6)
Therapeutic relationship HAS-Patient: mean (SD) [range]	8.0 (1.7) [0–10]

### Association between patient and clinician ratings of the therapeutic relationship

Patient and clinician ratings were weakly correlated with each other, r_s_ = 0.13 (p = 0.004).

### Length of time in treatment and the therapeutic relationship

Length of time in treatment was not associated with patient ratings (r_s_ = −0.12, p = 0.79) or clinician ratings (r_s_ = −0.00, p = 0.88) of the TR.

### Professional background of keyworkers and the therapeutic relationship

There were no significant differences across different keyworker professional backgrounds and patient (F = 1.34, p = 0.26) or keyworker ratings (F = 0.54, p = 0.70) of the TR.

### Therapeutic relationship and medication adherence

The distribution of adherence ratings was skewed (see Table 2) so it was transformed into a categorical variable with two categories: good adherence, i.e., ≥75% (N = 367) or average/poor adherence, i.e., <75% (N = 118).

**Table 2:** Distribution of Adherence Ratings.

Adherence to antipsychotic medication	Percentage
Good (>75%)	75.7%
Average (25–75%)	20.2%
Poor (<25%)	4.1%

In the average patient (see Table 3), for each unit increase in clinician rated TR score, the odds ratio of good compliance was increased by 65.9% (95% CI: 34.6% to 104.5%). A lesser increase of 20.8% (95% CI: 4.4% to 39.8%) in the odds ratio for good compliance was observed per unit increase in patient rated TR score. There was a small negative association between symptoms and adherence: OR = 0.894 (95% CI: 0.971 to 0.996). As country did not make a significant contribution to the model, it was excluded in the final model (Wald test χ_5_^2^ = 5.86, p = 0.32).

**Table 3:** Associations between Therapeutic Relationship and Adherence to Antipsychotic Medication in a logistic regression model based on 466 patients.

	Adherence to Antipsychotics		
	Adjusted odds ratio	95% CI	p
Patient rating of relationship	1.21	1.04 to 1.40	p = 0.017
Keyworker rating of relationship	1.66	1.35 to 2.05	p<0.001
Symptoms	0.98	0.97 to 0.99	p = 0.014

Among the patients receiving depot medication, 22% had poor adherence and 78% had good adherence. In this depot subgroup (see Table 4), for each unit increase in clinician rated relationship score, the odds ratio of good compliance was statistically significantly increased by 50.9% (95% CI: 1.01 to 2.25). For each unit increase in patient rated relationship score, the odds ratio of good compliance was increased by 34.8% (95% CI: 0.95 to 1.90) which was significant at p = 0.09.

**Table 4:** Associations between Therapeutic Relationship and Adherence to Antipsychotic Medication in depot subgroup in a logistic regression model based on 90 patients.

	Adherence to Antipsychotics		
	Adjusted odds ratio	95% CI	p
Patient rating of relationship	1.35	0.95 to 1.90	p = 0.090
Keyworker rating of relationship	1.51	1.01 to 2.25	p = 0.042
Symptoms	0.98	0.95 to 1.01	p = 0.279

To give some indication of the clinical relevance, the adherence of patients with better and poorer keyworker ratings of the TR was compared descriptively. This was done using 7 as a cutoff point as in previous studies [23]: above 7 on a satisfaction based measure indicates a better relationship while below 7 indicates a poorer relationship. If keyworkers rated the relationship with their patients less highly (<7 on the HAS), 42% of their patients had poor adherence compared to 17% if keyworkers rated the relationship more highly (≥7 on the HAS).

## Discussion

There are two main findings from this study. Firstly, the TR is associated with adherence to, not just attitudes towards, antipsychotic medication in the treatment of schizophrenia. Secondly, although only weakly correlated with each other, both patient and clinician perspectives of the TR are independently associated with adherence. In the depot subgroup, where the assessment of adherence is more objective and hence more reliable, the clinician's perspective remained significantly associated with adherence whereas the patient's perspective was significant at the 10% level.

With over 450 patients, this is the largest study to date to investigate the association between the TR in the treatment of schizophrenia and adherence to antipsychotic medication. It used the same assessment instruments in community mental healthcare settings across six European countries. The associations between the TR and adherence were seen after adjusting for symptom levels and possible clustering effects of patients treated by the same clinician and were not influenced by country. A limitation is that adherence was, in some cases, assessed by the clinician [29] who also rated their relationship with the patient. It should be considered that a clinician's ratings of the TR and medication adherence may not be independent of each other, i.e., their TR ratings are influenced by their estimations of medication adherence and their estimations of adherence may be influenced by the relationship they have with the patient. However, the assessment of adherence was based on collateral information from depot, supervised drug intake, drug tests and other clinicians (psychiatrist, general practitioner, pharmacist) and informal carers in the majority of cases. Moreover, the association between TR and adherence held true in a sub-sample of patients on depot medication where the assessment of adherence is objective and independent of any potential rater bias.

The sample is not necessarily representative for all patients with schizophrenia in community mental health care. Most patients had been in treatment for many years and were, on the whole, well engaged in treatment. Those who agreed to participate in the study, both patients and clinicians, may have had better TRs. Thus, there may have been a selection bias, including fewer patients with shorter treatment histories and poorer TRs. However, the TR ratings reported by patients (mean 8.0) in the current study are similar to those reported in other studies, e.g. a mean of 8.1 in outpatients with schizophrenia [34]. Moreover, length of time in treatment was not associated with the quality of the TR from either patient or clinician perspective. Finally, the associations are cross-sectional so causal relationships may not be inferred.

Patient and clinician ratings of the TR were only weakly correlated with each other, which in other studies has also been found in relation to needs for care [28]. It may be seen as intriguing that, despite the fact that patient and clinician perspectives on the TR are only weakly inter-correlated, both are independently associated with adherence. Hence, each perspective must be capturing some distinctive aspects. The patient's rating may be tapping into a subjective assessment of the social and personal experience of the relationship with their clinician. If they get along well with their clinician, they may be more willing to follow the clinician's advice on treatment. Clinicians have different views of the TR with a given patient. They may compare it to relationships with other patients and consider how the patient is functioning more generally. In turn, how well a patient is functioning may coincide, to some degree, with adherence to treatment.

In the current study, the clinician's perspective had a somewhat stronger association with adherence than the patient's perspective. This is in contrast to psychotherapy, where the patient's perspective appears to be most strongly related to outcome [30]. It is, however, consistent with other findings in psychiatry, which have measured both the patient's and clinician's perspectives. These studies suggest that the clinician's perspective may be more strongly related to outcome in complex psychiatric treatment of depression [17] and schizophrenia, psychosis or major affective disorder [7], [31]. It may be the case that clinicians rate the TR higher when patients adhere to their recommendations, i.e., they view their relationship with these patients more positively because they are more adherent. On a speculative note, this may also be related to the degree to which treatment is oriented to the needs of the patient as identified by themselves, which is stronger in psychotherapy, versus the needs of the patient as identified by the service, which is stronger in psychiatric treatment of patients with severe mental illness.

As mentioned above, the findings do not imply causality and could be interpreted in different ways. A better TR may lead to better adherence or better adherence may lead to a better TR or both. Future prospective studies with first episode samples would help to disentangle the direction of the effect. The associations might also be explained by other factors. One possibility is that those patients who form better relationships with their clinicians do so because they *can* and will also do better on a range of outcomes, in this case, more likely to adhere to medication. This might be an index of their individual potential rather than anything to do with the potential of the relationship *per se* to influence treatment outcomes. Bentall et al. [32] examined whether the TR indirectly mediates or has a direct causal influence on outcome in a large trial of cognitive behaviour therapy for psychosis. They found that the relationship had a direct causal influence on outcome, which was not explained by other factors influencing patients' potential to form a good TR.

Future studies might explore how to achieve better TRs. Little is known about what makes good relationships and how they might be improved, with a few intervention studies showing promising results [21], [33]. In the light of the current findings, studies developing and testing interventions that focus on collaboration in relationships and specifically on talk about medication may be indicated.

### Conclusion

In conclusion, the current findings suggest that better TRs between patients with schizophrenia and their clinicians in community care are important for adherence to antipsychotic medication. Furthermore, patient and keyworker perspectives on the relationship are not the same and both are independently associated with adherence.
